# Pro to AI/metaverse implementation: a review on the potential of metaverse in psychiatry

**DOI:** 10.1192/j.eurpsy.2024.137

**Published:** 2024-08-27

**Authors:** L. F. Fontenelle

**Affiliations:** Department of Psychiatry and Legal Medicine, Federal University of Rio de Janeiro (UFRJ) and D’Or Institute for Research and Education (IDOR), Rio de Janeiro, Brazil

## Abstract

**Abstract:**

The metaverse, a term first employed in Neal Stephenson’s 1992 novel “Snow Crash”, is a digital environment delivered via artificial intelligence in which multiple users can use avatars to engage in social, economic and cultural activities. Broadly speaking, metaverse encompasses technologies as diverse as augmented reality (AR), “lifelogging” (smart watches, smart phones and other wearables), “mirror” worlds (e.g. Google Earth, Waze, …) and virtual reality (VR). There is a pressing need to understand the potential of metaverse for medicine in general and psychiatry in particular. The therapeutic use of VR technologies is already a reality in clinical practice, particularly in terms of online treatments and exposure and response prevention for anxiety disorders, obsessive-compulsive and related disorders, and trauma-related disorders. Avatar integrated therapies may increase treatment seeking via anonymity, decrease in physical and communication barriers, and facilitation of expression. In terms of research, the metaverse allows manipulation of the therapeutic environment in order to answer specific questions.

**Disclosure of Interest:**

None Declared

